# Nationwide outcomes of endovascular thoracoabdominal aortic aneurysm repair

**DOI:** 10.1093/bjs/znag035

**Published:** 2026-03-26

**Authors:** Harry H Y Yu, Giuseppe Asciutto, Nuno V Dias, Anders Wanhainen, Angelos Karelis, Björn Sonesson, Kevin Mani

**Affiliations:** Department of Surgical Sciences, Section of Vascular Surgery, Uppsala University, Uppsala, Sweden; Department of Surgical Sciences, Section of Vascular Surgery, Uppsala University, Uppsala, Sweden; Vascular Centre, Department of Thoracic Surgery and Vascular Diseases, Skåne University Hospital, Malmö, Sweden; Department of Clinical Sciences Malmö, Lund University, Malmö, Sweden; Department of Surgical Sciences, Section of Vascular Surgery, Uppsala University, Uppsala, Sweden; Department of Diagnostics and Intervention, Umeå University, Umeå, Sweden; Vascular Centre, Department of Thoracic Surgery and Vascular Diseases, Skåne University Hospital, Malmö, Sweden; Department of Clinical Sciences Malmö, Lund University, Malmö, Sweden; Vascular Centre, Department of Thoracic Surgery and Vascular Diseases, Skåne University Hospital, Malmö, Sweden; Department of Clinical Sciences Malmö, Lund University, Malmö, Sweden; Department of Surgical Sciences, Section of Vascular Surgery, Uppsala University, Uppsala, Sweden

## Abstract

**Background:**

Complex endovascular repair has emerged as a minimally invasive alternative for thoracoabdominal aortic aneurysms (TAAAs). The aim of this study was to assess the short- and mid-term outcomes of endovascular TAAA repair in Sweden.

**Methods:**

All endovascular TAAA repairs performed during 2018–2023 were identified in the Swedish Vascular (Swedvasc) Registry. Patient characteristics, operative details, and outcomes were analysed. The primary outcome was survival; secondary outcomes included complications and thoracoabdominal aortic life-altering events (TALEs). Predictors of mid-term outcomes were assessed in multivariable analysis.

**Results:**

Some 339 patients were treated for TAAAs, which included 476 repairs (366 elective repairs and 110 emergency repairs) across 11 centres (94% concentrated in 5 centres). Elective patients (235 patients; mean diameter 64 mm) had 30-day, 90-day, and 1-year mortality of 2.6%, 3.5%, and 12.4% respectively. Major complications, vascular complications, and TALEs occurred in 16.2%, 9.8%, and 14% respectively. The use of prophylactic spinal drainage declined over time (from 68% to 14%) and the incidence of spinal cord ischaemia declined over time (from 20% to 2.3%). Mean(s.e.) 1-year and 4-year Kaplan–Meier survival estimates were 89.3%(2.1%) and 73.5%(3.7%) respectively. Emergency patients (104 patients; mean diameter 73 mm) had 30-day, 90-day, and 1-year mortality of 12.5%, 17.6%, and 26.9% respectively. Major complications, vascular complications, and TALEs occurred in 20.2%, 12.5%, and 24% respectively. Mean(s.e.) 1-year and 4-year Kaplan–Meier survival estimates were 75.2%(4.3%) and 56.5%(6.2%) respectively. Perioperative myocardial infarction was the strongest predictor of 1-year mortality after both elective and emergency repair.

**Conclusion:**

Endovascular TAAA repair in Sweden is associated with significant complication rates, but acceptable mid-term survival. Further research should focus on perioperative refinements, especially with regard to preserving organ function.

## Teaser

A decreasing trend of use of prophylatic spinal drainage for endovascular thoracoabdominal aortic aneurysm repair, with a corresponding decreasing incidence of spinal cord ischaemia, was observed during the study interval, 2018–2023.

## Introduction

Thoracoabdominal aortic aneurysm (TAAA) repair is very challenging. Traditional open surgical repair (OSR) poses significant perioperative risks, including spinal cord ischaemia (SCI), renal failure, and cardiopulmonary complications, as well as high perioperative mortality^1^. Many TAAA patients are old and have cardiopulmonary co-morbidities, markedly increasing the risks of mortality and complications after OSR. Patients may therefore be excluded from open surgical treatment due to excessive operative risk.

The advent of advanced endovascular fenestrated and branched techniques has revolutionized the management of TAAAs. Endovascular repair is a less invasive alternative that reduces operative trauma, shortens the length of hospital stay, and may improve patient outcomes^2^. Procedure advances, including fully percutaneous approaches, image-fusion guidance, staged repair strategies, and the use of steerable sheaths, have contributed to making endovascular TAAA repair an increasingly feasible option. Although endovascular repair is recommended as the first-line approach for patients with TAAAs, high-quality data regarding short- and mid-term outcomes of endovascular TAAA repair are sparse and the occurrence of complications such as stroke, SCI, and renal failure remains a significant concern^3^.

The aim of this study was to assess the outcomes of endovascular repair for TAAAs for patients in Sweden.

## Methods

This study used data from the Swedish National Registry for Vascular Surgery (Swedvasc) and is reported in accordance with the STROBE guidelines^4^. Ethics approval was obtained (Dnr 2021-0449), with the need for individual consent waived due to the retrospective character of the analysis. The study complied with the Declaration of Helsinki.

### Data source and study cohort

The Swedvasc Registry was created in 1987 and was updated in 2018 with regard to the aortic disease module. It has been extensively verified, with >90% external and internal validities for aortic procedures^5,6^. Survival data are automatically updated in the Swedvasc Registry through cross-linkage with the Swedish Population Register, ensuring no loss to follow-up for this endpoint^7^.

All patients who underwent endovascular treatment for TAAAs between June 2018 and December 2023 were identified. For staged procedures, subsequent procedures until May 2024 were also captured and, when required, further communication with each centre was established to ascertain the completeness of stages. Procedures for dissection were not included in this analysis, as dissection repair is registered in a separate part of the aortic module in the Swedvasc Registry. Data were analysed and are reported on a patient basis for those who underwent multiple procedures as part of staged procedures. Perioperative complications and deaths occurring after all stages of the operations were counted cumulatively on a patient basis. Both elective and emergency repairs were included.

### Clinical and outcome variables

Baseline characteristics, including patient demographics, co-morbidities, and aortic pathology characteristics, were analysed.

The following preoperative co-morbidities are recorded in the Swedvasc Registry and were assessed in this study: hypertension (treated medically); diabetes mellitus (treated by diet, oral hypoglycaemic agents, or insulin); pulmonary disease (any pre-existing diagnosis of pulmonary pathology); smoking history; cerebrovascular disease (including stroke, transient ischaemic attack, and cerebral haemorrhage); chronic kidney disease (serum creatinine >150 mmol/l or active renal replacement therapy); and heart disease (history of angina, myocardial infarction, heart failure, coronary artery bypass surgery, or percutaneous coronary intervention). The Glasgow aneurysm score (GAS; score = age in years + 7 for heart disease + 10 for cerebrovascular disease + 14 for chronic renal disease + 17 for circulatory shock (in emergency repair)) was calculated to adjust for individual risk profiles^8^.

Elective and emergency procedures were analysed separately. All aneurysms were classified anatomically in the Swedvasc Registry according to Safi’s modification of the Crawford classification system^9,10^. For this analysis, aneurysms were divided into three groups: extent II, extent IV, and others (that is extent I, extent III, and extent V).

Perioperative complications, that is complications within 30 days, were classified as major complications and vascular complications. Major complications included stroke, myocardial infarction, SCI (defined as any motor or sensory deficit in the lower limbs within 30 days, that is clinically classified as caused by spinal cord ischaemic insult), renal failure (defined as the need for renal replacement therapy at 30 days), multiorgan failure, bowel ischaemia, abdominal compartment syndrome, prolonged ICU stay (>5 days), and reoperation due to bleeding. Vascular complications included type I/III endoleaks, extremity ischaemia, distal embolism, graft occlusion, graft infection, and branch occlusion. Thoracoabdominal aortic life-altering events (TALEs; including perioperative mortality, dialysis, paralysis, and/or stroke, together forming a composite outcome) at 30 days were also analysed^11^.

The results of the study are reported in accordance with the reporting standards for complex aortic procedures wherever appropriate^12^.

### Statistical analysis

Categorical and dichotomous variables are presented as *n* (%). Continuous variables are presented as mean(s.d.) for normally distributed variables and as median (interquartile range (i.q.r.)) for non-normally distributed variables. Differences in demographic and outcome variables were tested for significance using Fisher’s exact test for categorical variables and Student’s *t* test or the Mann–Whitney *U* test for continuous variables. Univariable analysis by Cox regression was used to identify possible risk factors (including patient demographics, aneurysm characteristics, and perioperative complications) for TALEs and 1-year mortality. Factors with *P* < 0.200 were included in a multivariable logistic regression analysis. Survival was estimated using Kaplan–Meier analysis. All tests were two-sided. *P* < 0.050 was considered statistically significant. Statistical analysis was performed using SPSS^®^ (IBM, Armonk, NY, USA; Statistics, version 28).

## Results

Some 7279 aortic operations performed at 28 Swedish centres were recorded in the Swedvasc Registry during the study interval, with 339 patients (235 elective patients and 104 emergency patients) being treated for TAAAs, which included 476 endovascular procedures (366 elective procedures and 110 emergency procedures). These endovascular TAAA procedures were performed at 11 centres, with 5 centres performing 94.4% of the procedures. As a comparison, 47 open repair procedures for TAAAs were performed at eight vascular centres, with 66% being performed at one centre. The rate of endovascular repair during the study interval was as follows: 2018, 83.7%; 2019, 81.5%; 2020, 90.5%; 2021, 86.1%; 2022, 87.3%; and 2023, 96.9%. See *Table 1* for the baseline demographics of the 339 patients.

**Table 1:** Baseline demographics of patients who underwent endovascular TAAA repair in Sweden over a 6-year interval, 2018–2023

	Elective				Emergency			
	Extent II (*n* = 76)	Extent IV (*n* = 70)	Others (*n* = 89)	*P*	Extent II (*n* = 31)	Extent IV (*n* = 31)	Others (*n* = 42)	*P*
**Sex**				0.008				0.021
Male	38 (50)	52 (74.3)	51 (57.3)		14 (45.2)	24 (77.4)	22 (52.4)	
Female	38 (50)	18 (25.7)	38 (42.7)		17 (54.8)	7 (22.6)	20 (47.6)	
Age (years), mean(s.d.)	71.0(7.2)	74.0(6.6)	71.7(8.2)	0.038	71.6(7.7)	75.4(5.0)	74.3(7.3)	0.083
Hypertension	63 (82.9)	58 (82.9)	74 (83.1)	0.965	25 (80.6)	25 (80.6)	35 (83.3)	0.811
Heart disease	27 (35.5)	26 (37.1)	20 (22.5)	0.063	12 (38.7)	14 (45.2)	16 (38.1)	0.811
Diabetes	6 (7.9)	7 (10.0)	12 (13.5)	0.244	1 (3.2)	5 (16.1)	5 (11.9)	0.189
**Smoking**				0.063				0.112
Never	10 (13.2)	15 (21.4)	5 (5.6)		4 (12.9)	4 (12.9)	5 (11.9)	
Ex-smoker	33 (43.4)	29 (41.4)	41 (46.1)		10 (32.3)	10 (32.3)	10 (23.8)	
Active	12 (15.8)	14 (20.0)	15 (16.9)		9 (29.0)	5 (16.1)	8 (19.0)	
Unknown	21 (27.6)	12 (17.1)	28 (31.5)		8 (25.8)	12 (38.7)	19 (45.2)	
Lung disease	21 (27.6)	24 (34.3)	31 (34.8)	0.335	11 (35.5)	7 (22.6)	13 (31.0)	0.521
Stroke	6 (7.9)	5 (7.1)	10 (11.2)	0.439	3 (9.7)	5 (16.1)	5 (11.9)	0.736
Chronic kidney disease	9 (11.8)	14 (20.0	16 (18.0)	0.311	4 (12.9)	8 (25.8)	9 (19.0)	0.431
History of aortic repair	43 (56.6)	21 (30.0)	44 (49.4)	0.003	11 (35.5)	10 (32.3)	16 (38.1)	0.790
Connective tissue disease	6 (7.9)	1 (1.4)	5 (5.6)	0.150	1 (3.2)	0 (0.0)	2 (4.8)	0.321
GAS, mean(s.d.)	75.6(9.0)	80.1(9.4)	77.0(11.2)	0.023	81.5(13.5)	83.7(10.9)	83.2(10.9)	0.717
Maximal diameter (mm), mean(s.d.)	67.1(8.1)	62.6(8.8)	62.7(8.5)	0.001	73.2(11.8)	70.2(20.3)	72.1(24.1)	0.834

Values are *n* (%) unless otherwise indicated. TAAA, thoracoabdominal aortic aneurysm; GAS, Glasgow aneurysm score.

Among the 235 patients who underwent elective endovascular TAAA repair, 22 patients had extent I TAAAs, 76 patients had extent II TAAAs, 46 patients had extent III TAAAs, 70 patients had extent IV TAAAs, and 21 patients had extent V TAAAs. Most patients (133 patients (56.6%)) underwent staged repairs (extent II 59.2%, extent IV 21.4%, and others 52.8%; *P* < 0.001). A total of 423 visceral vessels (branch = 242, fenestration = 175, chimney = 1, and others = 5) and 427 renal vessels (branch = 175, fenestration = 247, and others = 5) were revascularized.

Among the 104 patients who underwent emergency endovascular TAAA repair, 8 patients had extent I TAAAs, 31 patients had extent II TAAAs, 19 patients had extent III TAAAs, 31 patients had extent IV TAAAs, and 15 patients had extent V TAAAs. A quarter of the patients (26 of 104 patients) had ruptured aneurysms, with 14 patients suffering from circulatory shock and 3 patients suffering from loss of consciousness. A total of 150 visceral vessels (branch = 98, fenestration = 41, chimney = 2, and others = 9) and 142 renal vessels (branch = 96, fenestration = 44, and chimney = 2) were revascularized.

### Survival

The median follow-up duration was 24.8 (i.q.r. 11.1–43.5) months in the elective group and 23.2 (i.q.r. 6.3–40.5) months in the emergency group. When excluding 90-day mortality (231 elective patients and 86 emergency patients), the median follow-up for those surviving the perioperative interval was 26.0 (i.q.r. 11.6–45.4) months in the elective group and 28.3 (i.q.r. 13.4–42.9) months in the emergency group.

**Fig. 1:**
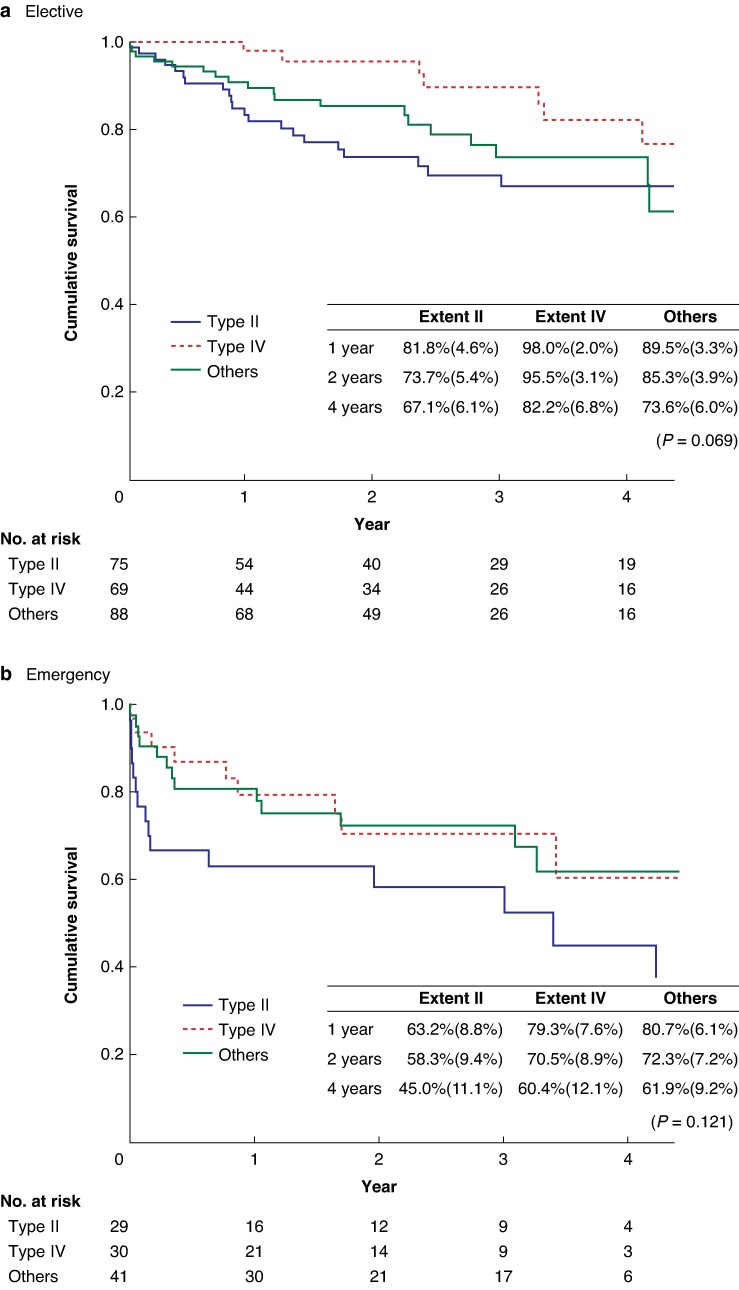
Survival (estimated using Kaplan–Meier analysis) after endovascular repair for TAAAs in Sweden over a 6-year interval, 2018–2023 **a** Elective. **b** Emergency. Survival estimates at 1 year, 2 years, and 4 years are presented in the embedded tables as mean(s.e.) values. TAAAs, thoracoabdominal aortic aneurysms.

In the elective group, 30-day survival was 97.4% (95% c.i. 94.4% to 99.0%), that is the 30-day mortality rate was 2.6% (*Table 2*). Survival was 96.5% (95% c.i. 93.2% to 98.3%) at 90 days and 87.6% (95% c.i. 82.2% to 91.6%) at 1 year. Comparing survival between patients with aneurysms of different extents, survival for patients with extent IV TAAAs was significantly higher at 1 year (extent II 79.4%, extent IV 97.9%, and others 88.6%; *P* = 0.006), but not at 30 days (extent II 96.1%, extent IV 100%, and others 96.6%; *P* = 0.114) or 90 days (extent II 94.7%, extent IV 100%, and others 95.5%; *P* = 0.056). Mean(s.e.) estimated survival was 89.3%(2.1%) at 1 year and 73.5%(3.7%) at 4 years (*Fig. 1a*).

**Table 2:** Thirty-day mortality and TALEs per year for patients who underwent elective endovascular TAAA repair in Sweden over a 6-year interval, 2018–2023

	2018	2019	2020	2021	2022	2023	*P*
**Elective**							
Thirty-day mortality	8.0	2.2	0.0	0.0	4.3	2.3	0.356
TALEs	28.0	11.1	13.9	15.4	12.8	9.3	0.383
**Emergency**							
Thirty-day mortality	9.1	12.5	9.5	13.0	18.2	10.5	0.668
TALEs	18.2	37.5	23.8	21.7	27.3	21.1	0.930

Values are % unless otherwise indicated. TALEs, thoracoabdominal aortic life-altering events; TAAA, thoracoabdominal aortic aneurysm.

In the emergency group, 30-day survival was 87.5% (95% c.i. 79.6% to 92.7%), that is the 30-day mortality rate was 12.5%. Survival was 82.4% (95% c.i. 73.7% to 88.6%) at 90 days and 73.1% (95% c.i. 63.3% to 81.1%) at 1 year. Comparing survival between patients with aneurysms of different extents, the survival rates were as follows: 30 days—extent II 77.4%, extent IV 93.5%, and others 90.5% (*P* = 0.119); 90 days—extent II 67.7%, extent IV 90.0%, and others 87.8% (*P* = 0.036); and 1 year—extent II 60.7%, extent IV 80.8%, and others 81.1% (*P* = 0.073). Mean (s.e.) estimated survival was 75.2%(4.3%) at 1 year and 56.5%(6.2%) at 4 years (*Fig. 1b*).

Emergency patients with ruptured aneurysms had significantly worse survival compared with emergency patients with non-ruptured aneurysms: at 30 days, ruptured 69.2% and non-ruptured 93.6% (*P* = 0.003); at 90 days, ruptured 60% and non-ruptured 89.6% (*P* = 0.002); and at 1 year, ruptured 56% and non-ruptured 81.8% (*P* = 0.014).

In the elective group, the mean(s.d.) GAS was 77(10.2) (range 36–105) and a GAS >85 was associated with higher 1-year mortality (GAS ≤85 = 9.2% and GAS >85 = 20.5%; *P* = 0.047). In the emergency group, the mean(s.d.) GAS was 82.9(11.6) (range 55–122). A trend similar to that noted in the elective group was noted in the emergency group, though it did not reach statistical significance (GAS ≤85 = 33.8% and GAS >85 = 47.8%; *P* = 0.171).

### Perioperative complications

In the elective group, there were 44 perioperative major complications captured in 38 patients (16.2%) and 24 vascular complications in 23 patients (9.8%). A trend of higher incidence of major complications among extent II TAAAs was observed (extent II 22.4%, extent IV 12.9%, and others 13.5%; *P* = 0.133). The overall rate of TALEs after elective repair was 14% (extent II 22.4%, extent IV 7.1%, and others 12.4%; *P* = 0.026) (*Table 2* and *Table 3*).

**Table 3:** Major and vascular complications within 30 days for patients who underwent endovascular TAAA repair in Sweden over a 6-year interval, 2018–2023

	Elective				Emergency			
	Extent II (*n* = 76)	Extent IV (*n* = 70)	Others (*n* = 89)	*P*	Extent II (*n* = 31)	Extent IV (*n* = 31)	Others (*n* = 42)	*P*
**Major complications**								
Stroke	3 (3.9)	1 (1.4)	0 (0.0)	0.053	2 (6.5)	1 (3.2)	0 (0.0)	0.104
Myocardial infarction	1 (1.3)	1 (1.4)	1 (1.1)	0.908	0 (0.0)	1 (3.2)	0 (0.0)	0.295
SCI	10 (13.2)	3 (4.3)	6 (6.7)	0.122	3 (9.7)	2 (6.5)	4 (9.5)	0.130
Renal failure	6 (7.9)	1 (1.4)	4 (4.5)	0.155	3 (9.7)	5 (16.1)	2 (4.8)	0.265
Multiorgan failure	2 (2.6)	0 (0.0)	2 (2.2)	0.236	2 (6.5)	2 (6.5)	2 (4.8)	0.749
Bowel ischaemia	2 (2.6)	1 (1.4)	2 (2.2)	0.870	1 (3.2)	2 (6.5)	0 (0.0)	0.172
Abdominal compartment syndrome	0 (0.0)	0 (0.0)	1 (1.1)	0.259	0 (0.0)	0 (0.0)	0 (0.0)	NA
Prolonged ICU stay (>5 days)	5 (6.6)	2 (2.9)	4 (4.5)	0.553	5 (16.1)	2 (6.5)	3 (7.1)	0.223
Reoperation due to bleeding	0 (0.0)	2 (2.9)	2 (2.2)	0.200	2 (6.5)	0 (0.0)	2 (4.8)	0.224
Total major complications	17 (22.4)	9 (12.9)	12 (13.5)	0.133	8 (25.8)	6 (19.4)	10 (23.8)	0.822
**Vascular complications**								
Type I/III endoleaks	3 (3.9)	4 (5.7)	4 (4.5)	0.875	3 (9.7)	0 (0.0)	2 (4.8)	0.116
Extremity ischaemia	1 (1.3)	1 (1.4)	0 (0.0)	0.347	0 (0.0)	2 (6.5)	0 (0.0)	0.085
Distal embolism	3 (3.9)	0 (0.0)	3 (3.4)	0.114	0 (0.0)	2 (6.5)	1 (2.4)	0.234
Graft occlusion	2 (2.6)	0 (0.0)	0 (0.0)	0.074	0 (0.0)	1 (3.2)	0 (0.0)	0.295
Graft infection	0 (0.0)	0 (0.0)	1 (1.1)	0.259	3 (9.7)	0 (0.0)	0 (0.0)	0.020
Branch occlusion	1 (1.3)	2 (2.9)	0 (0.0)	0.191	0 (0.0)	1 (3.2)	0 (0.0)	0.295
Total vascular complications	8 (10.5)	7 (10.0)	8 (9.0)	0.739	6 (19.4)	4 (12.9)	3 (7.1)	0.120
TALEs	17 (22.4)	5 (7.1)	11 (12.4)	0.026	10 (32.3)	5 (16.1)	10 (23.8)	0.331

Values are *n* (%) unless otherwise indicated. TAAA, thoracoabdominal aortic aneurysm; SCI, spinal cord ischaemia; NA, not applicable; TALEs, thoracoabdominal aortic life-altering events.

In the emergency group, there were 21 perioperative major complications captured in 21 patients (20.2%) and 13 vascular complications in 13 patients (12.5%). The incidence of major complications according to aneurysm extent was as follows: extent II, 25.8%; extent IV, 19.4%; and others, 23.8% (*P* = 0.822). The incidence of vascular complications according to aneurysm extent was as follows: extent II, 19.4%; extent IV, 12.9%; and others, 7.1% (*P* = 0.120). The overall rate of TALEs after emergency repair was 24% (extent II 32.3%, extent IV 16.1%, and others 23.8%; *P* = 0.331).

### Elective staged procedures

Some 133 patients were registered as undergoing staged TAAA repair in the database. For 17 of these patients (12.8%), the aortic procedure registered in the Swedvasc Registry was not the first stage of the repair, for example the patient may have undergone a supra-aortic bypass, an iliac access operation, or a frozen elephant trunk procedure that was performed by a cardiothoracic surgeon(s) as the first stage of the repair and which was not captured in the database. Nine patients (6.8%) did not complete the expected staged operation, due to mortality after an early stage for one patient and due to perioperative complications after an early stage for three patients; for five patients, the cause of lack of completion of repair could not be elucidated.

Among the 102 patients for whom multiple stages were identified, most underwent endovascular TAAA repair in two stages (94 patients (92.2%)), 7 patients (6.9%) underwent endovascular TAAA repair in three stages, and 1 patient (1.0%) underwent endovascular TAAA repair in four stages. The median duration between stages was 44 (i.q.r. 27–77) days. Mortality was observed in one patient (0.9%) after the first stage and in two patients (1.8%) during the later stages. TALEs were observed in 6 patients (5.5%) after the first stage and 14 patients (12.5%) during the later stages.

### SCI

**Fig. 2:**
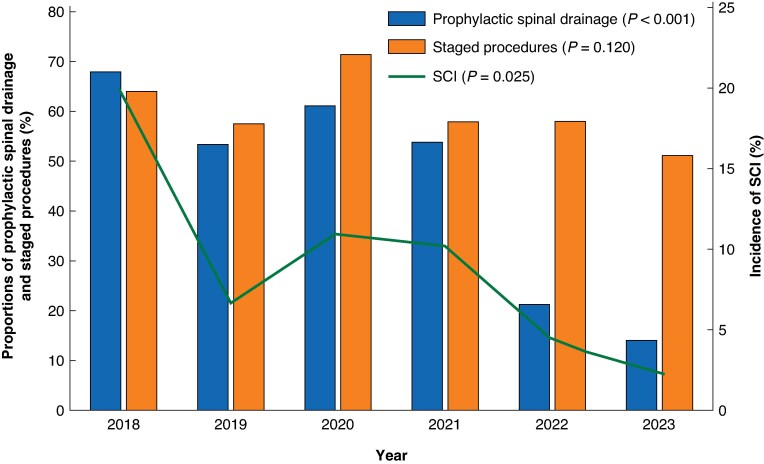
Annual proportions of prophylactic spinal drainage and staged procedures and incidence of SCI for elective endovascular TAAA repair in Sweden over a 6-year interval, 2018–2023 SCI, spinal cord ischaemia; TAAA, thoracoabdominal aortic aneurysm.

In the full cohort, the incidence of SCI was 8.1%. A significant reduction in the incidence of SCI among the elective group was observed over the study interval (from 20% in 2018 to 2.3% in 2023; *P* = 0.041). A simultaneous decrease in the use of prophylactic spinal drainage was observed (from 68% in 2018 to 14% in 2023; *P* < 0.001), while the proportion of staged procedures during the study interval remained stable (*Fig. 2*). Overall, the proportion of staged procedures was 56.6% (extent II 77.6%, extent IV 30%, and others 59.6%; *P* = 0.120). Comparing between extents of aneurysm, extent II aneurysms had a trend of increased risk of SCI (extent II 13.2%, extent IV 4.3%, and others 6.7%; *P* = 0.176).

In the emergency group, no clear time trends in the use of prophylactic spinal drainage (mean 28.8% and range 6.7–36.7%; *P* = 0.053) or the incidence of SCI (mean 8.7% and range 0–25%; *P* = 0.604) were observed. The incidence of SCI according to aneurysm extent in the emergency group was as follows: extent II, 9.7%; extent IV, 6.5%; and others, 9.5% (*P* = 0.130).

### Risk factors for TALEs and mortality

Factors associated with TALEs at 30 days (*Table 4*) and mortality at 1 year (*Table 5*) were identified. For TALEs, there was a trend of reduced incidence during the study interval. Furthermore, the presence of an extent II TAAA was a risk factor for TALEs, while extent IV aneurysms and history of aortic repair were associated with a reduced incidence of TALEs.

**Table 4:** Risk factors for 30-day TALEs by Cox regression for patients who underwent endovascular TAAA repair in Sweden over a 6-year interval, 2018–2023

Factors	Elective				Emergency			
	Univariable analysis		Multivariable analysis		Univariable analysis		Multivariable analysis	
	RR (95% c.i.)	*P*	RR (95% c.i.)	*P*	RR (95% c.i.)	*P*	RR (95% c.i.)	*P*
Age >80 years	1.75 (0.67,4.58)	0.26			1.85 (0.73,4.73)	0.20	2.07 (0.74,5.78)	0.17
Male	0.82 (0.41,1.64)	0.58			0.67 (0.31,1.48)	0.33		
Hypertension	1.53 (0.54,4.37)	0.43			0.68 (0.25,1.84)	0.45		
Heart disease	0.69 (0.30,1.60)	0.39			0.65 (0.28,1.50)	0.31		
Diabetes	1.46 (0.51,4.17)	0.48			1.22 (0.36,4.14)	0.75		
Smoking history	1.02 (0.92,1.13)	0.72			0.98 (0.88,1.09)	0.69		
Lung disease	1.55 (0.78,3.10)	0.21			1.13 (0.49,2.65)	0.77		
Stroke	0.58 (0.14,2.46)	0.46			1.11 (0.33,3.75)	0.87		
Chronic kidney disease	1.88 (0.85,4.20)	0.12	2.08 (0.92,4.67)	0.08	1.48 (0.58,3.73)	0.41		
History of aortic repair	0.58 (0.27,1.27)	0.17	0.41 (0.18,0.93)	0.03	0.78 (0.32,1.90)	0.59		
Connective tissue disease	0.05 (0,71.34)	0.41			1.51 (0.20,11.24)	0.69		
Extent II aneurysm	1.98 (1.00,3.93)	0.05	1.62 (1.18,2.23)	<0.01	1.80 (0.80,4.02)	0.16	2.26 (0.97,5.26)	0.06
Extent IV aneurysm	0.38 (0.15,0.99)	0.048	0.28 (0.10,0.80)	0.02	0.58 (0.22,1.57)	0.29		
Staged procedure	0.31 (0.05,1.89)	0.20	1.14 (0.52,2.47)	0.74				
Operation year	1.48 (1.09,2.01)	0.01	1.62† (1.18,2.23)	<0.01	1.26 (0.94,1.70)	0.13	1.17 (0.85,1.62)	0.34
Rupture					4.28 (1.88,9.74)	<0.01	4.60 (2.00,10.62)	<0.01
Loss of consciousness					1.26 (0.17,9.39)	0.82		
Circulatory shock					4.54 (1.92,10.74)	<0.01	3.94 (1.62,9.57)	<0.01

†Risk highest in 2018 (21.2%) and gradually decreased with time (12.1% in 2023). TALEs, thoracoabdominal aortic life-altering events; TAAA, thoracoabdominal aortic aneurysm; RR, relative risk.

**Table 5:** Risk factors for 1-year mortality by Cox regression for patients who underwent endovascular TAAA repair in Sweden over a 6-year interval, 2018–2023

Factors	Elective				Emergency			
	Univariable analysis		Multivariable analysis		Univariable analysis		Multivariable analysis	
	RR (95% c.i.)	*P*	RR (95% c.i.)	*P*	RR (95% c.i.)	*P*	RR (95% c.i.)	*P*
**Demographics**								
Age >80 years	1.99 (0.73,5.39)	0.18	2.97 (0.99,8.85)	0.05	2.34 (0.99,5.53)	0.05	3.90 (1.07,14.29)	0.04
Male	1.05 (0.45,2.46)	0.91			0.95 (0.42,2.17)	0.90		
Hypertension	2.15 (0.50,9.19)	0.30			0.38 (0.16,0.92)	0.03	0.27 (0.09,0.83)	0.02
Heart disease	1.07 (0.44,2.61)	0.89			0.45 (0.17,1.20)	0.11	0.53 (0.16,1.83)	0.32
Diabetes	1.10 (0.26,4.70)	0.90			0.30 (0.04,2.23)	0.24		
Smoking history	1.10 (0.99,1.23)	0.08	1.12 (0.99,1.27)	0.07	0.96 (0.85,1.07)	0.43		
Lung disease	0.57 (0.21,1.56)	0.28			0.79 (0.31,2.00)	0.62		
Stroke	2.08 (0.71,6.16)	0.18	2.63 (0.72,9.60)	0.14	0.67 (0.16,2.87)	0.59		
Chronic kidney disease	1.67 (0.62,4.53)	0.31			1.05 (0.39,2.83)	0.92		
History of aortic repair	1.02 (0.44,2.36)	0.97			0.82 (0.34,1.99)	0.66		
Connective tissue disease	0.69 (0.09,5.14)	0.72			1.47 (0.20,10.88)	0.71		
Extent II aneurysm	2.83 (1.21,6.63)	0.02	3.36 (1.33,8.48)	0.01	2.51 (1.11,5.69)	0.03	4.03 (1.35,12.03)	0.01
Extent IV aneurysm	0.14 (0.02,1.02)	0.05	0.13 (0.02,0.97)	0.05	0.63 (0.24,1.71)	0.37		
Staged procedure	1.20 (0.52,2.79)	0.67			7.09 (0.96,52.61)	0.06	4.82 (0.58,39.87)	0.15
Operation year	1.00 (0.75,1.34)	>0.99			1.26 (0.93,1.69)	0.13	0.94 (0.63,1.41)	0.77
Rupture					3.11 (1.37,7.06)	0.01	4.02 (1.34,12.10)	0.01
Loss of consciousness					0.05 (0,660.44)	0.53		
Circulatory shock					2.83 (1.11,7.20)	0.03	1.16 (0.28,4.76)	0.83
**Thirty-day complications**								
Stroke	0.05 (0,>9999)	0.68			8.52 (2.38,30.44)	<0.01	4.15 (0.63,27.39)	0.14
Myocardial infarction	6.79 (0.91,50.72)	0.06	12.38 (1.11,137.80)	0.04	4.50 (0.60,33.70)	0.14	29.66 (1.59,553.72)	0.02
SCI	1.01 (0.24,4.32)	0.99			1.24 (0.92,1.67)	0.16	0.60 (0.34,1.05)	0.08
Renal failure	5.85 (1.98,17.30)	<0.01	2.25 (0.48,10.62)	0.31	5.08 (2.07,12.42)	<0.01	6.80 (1.78,25.91)	0.01
Bowel ischaemia	7.33 (1.71,31.42)	0.01	9.94 (1.74,56.87)	0.01	1.49 (0.20,11.08)	0.70		
Reoperation due to bleeding	0.05 (0,>9999)	0.64			4.12 (1.22,13.96)	0.02	2.02 (0.33,12.25)	0.45
Type I/III endoleaks	0.92 (0.12,6.85)	0.94			3.28 (0.97,11.08)	0.06	2.00 (0.48,8.40)	0.35
Extremity ischaemia	4.47 (0.60,33.24)	0.14	4.11 (0.42,40.02)	0.22	2.63 (0.35,19.59)	0.35		
Distal embolism	1.65 (0.22,12.30)	0.62			1.49 (0.20,11.08)	0.70		
Graft occlusion	7.30 (0.98,54.46)	0.05	0 (0,>999)	0.98	0.05 (0,>999)	0.72		
Graft infection	0.05 (0,>9999)	0.82			1.20 (0.16,8.93)	0.86		
Branch occlusion	63.17 (6.57,607.24)	<0.01	>999 (0,>999)	0.97	0.05 (0,>9999)	0.72		

TAAA, thoracoabdominal aortic aneurysm; RR, relative risk.

In the elective group, multivariable analysis confirmed an extent II TAAA as a risk factor for 1-year mortality, while treatment due to an extent IV TAAA was associated with higher survival. The occurrence of perioperative myocardial infarction and the occurrence of perioperative bowel ischaemia were associated with an increased risk of 1-year mortality.

In the emergency group, a ruptured aneurysm and circulatory shock were associated with an increased incidence of TALEs. Age >80 years, rupture, and extent II aneurysms were associated with increased mortality at 1 year, while hypertension was identified as a protective factor. The occurrence of perioperative renal failure and the occurrence of perioperative myocardial infarction were also associated with an increased risk of mortality at 1 year.

In both the elective group and the emergency group, neither octogenarians (elective: OR 1.99 (95% c.i. 0.73 to 5.39); *P* = 0.176; and emergency: OR 2.34 (95% c.i. 0.99 to 5.53); *P* = 0.052) nor female patients (elective: OR 1.05 (95% c.i. 0.45 to 2.46); *P* = 0.907; and emergency: OR 0.95 (95% c.i. 0.42 to 2.17); *P* = 0.903) were associated with mortality up to 1 year.

## Discussion

Repair of TAAAs was first performed in the 1950s^12^. Coselli *et al*.^1^ reported the results of 3309 open TAAA repairs performed at a highly specialized expert centre between 1986 and 2014, with perioperative mortality of 6.2% and 12.2% for elective patients and emergency patients respectively, with an incidence of permanent SCI of 5.3% and an incidence of renal failure of 5.7%. Despite advancement in operative and medical approaches, high perioperative mortality for OSR (from 7.4% to 17.4%) has been confirmed in recent reports^11,13,14^. In an analysis of the Society of Thoracic Surgeons (STS) Adult Cardiac Surgery Database (ACSD), operative mortality for elective OSR for TAAAs was 11.7%, with an SCI rate of 6.0%, potentially better reflecting clinical outcomes outside of highly specialized centres^15^.

The development of more advanced endovascular techniques has changed the treatment of TAAAs dramatically. Significant technical development has taken place in endovascular TAAA repair in the past decade. Standardization of devices for TAAA repair with established platforms for off-the-shelf and custom-made stent grafts has resulted in more homogeneous surgical techniques. Important steps forward include the development of techniques for reduction of groin sheath size and use of steerable sheaths for both fenestrated and branched endovascular TAAA repairs. These developments may reduce ischaemic complications, as well as the risk of stroke associated with upper arm access^16,17^. These technical developments have also increased the possibility of total percutaneous TAAA repair. Image-fusion and routine use of hybrid operating rooms may further reduce operating time, as well as radiation and contrast use^18,19^. Additionally, strategies aiming to reduce SCI with staged procedures, as well as dedicated SCI protocols, are increasingly common in centres performing endovascular TAAA repair^20^. This has led to a progressive shift from OSR to endovascular repair. In Sweden, the vast majority of TAAA repairs are performed using endovascular techniques and the proportion was >90% in the final year of the present study. Such a trend has also been reported in other studies^2,21^.

The present study included the results of TAAA repairs in Sweden in the more recent era and thus the majority of the nation’s learning curve is not included. It showed a low 30-day mortality for elective patients who underwent endovascular repair for TAAAs. The 30-day mortality of 2.6% is comparable to that of previous single-centre studies (4.8%^22^ and 2.5%^23^) and the lowest among other nationwide or multicentre studies (6.5–10.8% in-hospital mortality)^2,11,15^. The incidences of SCI and renal failure of 8.1% and 4.7% respectively in elective patients and 8.7% and 9.6% respectively in emergency patients are comparable to those of other expert centres. Notably, endovascular TAAA repair in Sweden is primarily performed at highly specialized centres, with 94.4% of repairs being performed at five centres. These are all tertiary referral centres with established infrastructures (for example hybrid operating rooms with fusion technology) and know-how (experience of dedicated fenestrated and branched device platforms, and adjuncts such as steerable sheaths) for complex endovascular aortic repair^24^. The study interval, starting in 2018, reflects the modern and experienced practice of endovascular TAAA repair in Sweden.

Long-term survival rates after endovascular repair of 73.5% for elective patients and 56.5% for emergency patients at 4 years are comparable to those of other studies. Oderich *et al*.^23^ reported the results of 316 patients who underwent fenestrated-branched endovascular aneurysm repair for pararenal aneurysms or TAAAs using physician-modified endografts or company-manufactured devices, with a 3-year survival of 67.2–68.0%. Eagleton *et al*.^22^ reported a 5-year survival rate of 40% for 354 high-risk patients who underwent fenestrated and branched endovascular aneurysm repair for type II and III TAAAs between 2004 and 2013, and Khoury *et al*.^13^ reported a 10-year survival rate of 51% after endovascular repair for descending thoracic aortic aneurysms or TAAAs, with aortic-related mortality of 2.1%. Another study reported a 5-year survival rate of 55.3% after endovascular TAAA repair^11^. This can be compared with abdominal aortic aneurysm (AAA) repair, for which analysis of the Medicare-matched Society for Vascular Surgery (SVS) Vascular Quality Initiative (VQI) Vascular Implant Surveillance and Interventional Outcomes Network (VISION) database revealed 6-year survival rates of 64.4% and 58.8% for intact open and endovascular repair respectively^25^. In Sweden, based on a Swedvasc Registry study evaluating patients treated between 2010 and 2014, 5-year survival was reported to be 75.3% after intact AAA repair and 47.0% after ruptured AAA repair^26^. In the present study, a significant number of patients did not survive more than a year after endovascular TAAA repair. Better understanding of aortic pathology, as well as improved risk prediction tools, may assist in choosing patients who will truly benefit from TAAA repair in the long-term.

Complication rates after endovascular repair for TAAAs remained high. In particular, 22.6% of patients treated electively and 30.8% of patients treated as emergencies suffered from major or vascular complications. Furthermore, the incidence of TALEs was 14% for elective repairs and 24% for emergency repairs. This can be compared with an incidence of TALEs of 17.4% after endovascular TAAA repair in a multicentre population-based study from Ontario, Canada^11^, and an incidence of TALEs of 10% after extent I–III TAAA endovascular repair registered in the VQI registry between 2011 and 2022^27^. In the present study, a temporal trend of decreasing incidence of TALEs was observed for both elective and emergency repairs. This may be related to ongoing improvements in the management of this complex disease, but may also be the result of selection bias.

An interesting observation is the simultaneous reduced use of prophylactic spinal drainage and significant reduction in the incidence of SCI during the study interval, while the proportion of staged procedures remained unchanged. This is in line with previous reports of serious complications related to prophylactic spinal drainage^28,29^ and the latest trend appears to involve moving away from prophylactic spinal drainage, instead relying on timely detection of SCI, a dedicated SCI postoperative protocol (including maintaining higher mean arterial blood pressure, haemoglobin, oxygen saturation, and normal glucose level), and swift placement of therapeutic spinal drainage.

The GAS was used for this data set for score-adjusted analysis of outcomes. The Swedvasc Registry data did not contain adequate information for other scoring systems to be used for stratification. Patients with a GAS >85 suffered a high mortality rate of >20% at 1 year after elective repair. While a high GAS is not a contraindication to repair per se, this finding underlines the importance of proper selection of patients, considering both age and co-morbidities, to ensure that the patients exposed to extensive TAAA repair benefit from the procedure.

Despite the rapid evolution of endovascular techniques in the repair of complex aortic aneurysms, some major guidelines are indifferent in their recommendations regarding the preferred technique for TAAA repair, probably related to the timing of publication. The most recent American College of Cardiology and American Heart Association guideline (published in 2022) endorses open repair as the preferred therapy for patients with TAAAs who require intervention, due to the lack of US Food and Drug Administration (FDA)-approved devices for endovascular TAAA repair^30^. The latest European Society for Vascular Surgery (ESVS) guidelines (published in 2026) recommend endovascular repair as the primary choice for patients with TAAAs^3^. The American guideline also suggests prophylactic spinal drainage to reduce the incidence of SCI, while the European guidelines support selective, reactive use of CSF drainage over routine prophylaxis during extensive thoracic endovascular aortic repair. The present study showed a clear endovascular first-line approach to TAAA repair in Sweden, with very competitive outcomes in a national registry. Additionally, the incidence of SCI declined with less prophylactic spinal drainage, supporting the current ESVS recommendations.

There are several limitations to this study. Despite data being collected prospectively, the retrospective character of the analysis and the inherent risk of selection bias in registry-based analyses affect the generalizability of the results. While dissection repair is registered in a separate part of the aortic disease module of the Swedvasc Registry, there is a risk that a surgeon may input data as an aneurysm for a post-dissection aneurysm, although clear instructions during online registration aim to reduce this risk. Furthermore, it cannot be excluded that patients may have undergone planned staged procedures after the study interval. As the Swedvasc Registry exclusively collects data for patients undergoing surgery, no data are available for patients who are excluded from treatment. Follow-up after 30 days was not standardized and therefore data regarding reintervention (including endoleak or graft occlusion) beyond 30 days were not available for analysis. In addition, it was not possible to assess the cause of death of patients from the database. Due to a small number of patients and the risk of selection bias for the few patients undergoing OSR for TAAAs in Sweden, the outcomes of OSR were not analysed.

## Data Availability

Data are available upon reasonable request.
